# The causality of gut microbiota on onset and progression of sepsis: a bi-directional Mendelian randomization analysis

**DOI:** 10.3389/fimmu.2024.1266579

**Published:** 2024-04-18

**Authors:** Yuzheng Gao, Lidan Liu, Yuning Cui, Jiaxin Zhang, Xiuying Wu

**Affiliations:** Department of Anesthesia, ShengJing Hospital of China Medical University, Shenyang, Liaoning, China

**Keywords:** causal relationship, genetics, gut microbiota, Mendelian randomization, sepsis

## Abstract

**Background:**

Several observational studies have proposed a potential link between gut microbiota and the onset and progression of sepsis. Nevertheless, the causality of gut microbiota and sepsis remains debatable and warrants more comprehensive exploration.

**Methods:**

We conducted a two-sample Mendelian randomization (MR) analysis to test the causality between gut microbiota and the onset and progression of sepsis. The genome-wide association study (GWAS) summary statistics for 196 bacterial traits were extracted from the MiBioGen consortium, whereas the GWAS summary statistics for sepsis and sepsis-related outcomes came from the UK Biobank. The inverse-variance weighted (IVW) approach was the primary method used to examine the causal association. To complement the IVW method, we utilized four additional MR methods. We performed a series of sensitivity analyses to examine the robustness of the causal estimates.

**Results:**

We assessed the causality of 196 bacterial traits on sepsis and sepsis-related outcomes. Genus *Coprococcus2* [odds ratio (OR) 0.81, 95% confidence interval (CI) (0.69–0.94), *p* = 0.007] and genus *Dialister* (OR 0.85, 95% CI 0.74–0.97, *p* = 0.016) had a protective effect on sepsis, whereas genus *Ruminococcaceae UCG011* (OR 1.10, 95% CI 1.01–1.20, *p* = 0.024) increased the risk of sepsis. When it came to sepsis requiring critical care, genus *Anaerostipes* (OR 0.49, 95% CI 0.31–0.76, *p* = 0.002), genus *Coprococcus1* (OR 0.65, 95% CI 0.43–1.00, *p* = 0.049), and genus *Lachnospiraceae UCG004* (OR 0.51, 95% CI 0.34–0.77, *p* = 0.001) emerged as protective factors. Concerning 28-day mortality of sepsis, genus *Coprococcus1* (OR 0.67, 95% CI 0.48–0.94, *p* = 0.020), genus *Coprococcus2* (OR 0.48, 95% CI 0.27–0.86, *p* = 0.013), genus *Lachnospiraceae FCS020* (OR 0.70, 95% CI 0.52–0.95, *p* = 0.023), and genus *Victivallis* (OR 0.82, 95% CI 0.68–0.99, *p* = 0.042) presented a protective effect, whereas genus *Ruminococcus torques group* (OR 1.53, 95% CI 1.00–2.35, *p* = 0.049), genus *Sellimonas* (OR 1.25, 95% CI 1.04–1.50, *p* = 0.019), and genus *Terrisporobacter* (OR 1.43, 95% CI 1.02–2.02, *p* = 0.040) presented a harmful effect. Furthermore, genus *Coprococcus1* (OR 0.42, 95% CI 0.19–0.92, *p* = 0.031), genus *Coprococcus2* (OR 0.34, 95% CI 0.14–0.83, *p* = 0.018), and genus *Ruminiclostridium6* (OR 0.43, 95% CI 0.22–0.83, *p* = 0.012) were associated with a lower 28-day mortality of sepsis requiring critical care.

**Conclusion:**

This MR analysis unveiled a causality between the 21 bacterial traits and sepsis and sepsis-related outcomes. Our findings may help the development of novel microbiota-based therapeutics to decrease the morbidity and mortality of sepsis.

## 1 Introduction

Sepsis, one of the oldest and most elusive syndromes in medicine (1), is a critical global public health issue and a leading cause of morbidity and mortality worldwide (2). With the aging of the population leading to suppressed immunity, advances in medical care including immune-modulating medications, and the impact of global warming, sepsis is predicted to become an increasingly prevalent concern (3). Sepsis currently accounts for nearly 26% of all global deaths, resulting in more than 20 deaths per minute (4). The pathogenesis of sepsis is still not fully understood. Sepsis can be caused by infections stemming from viruses, fungi, or parasites, and non-immune alterations are known to contribute to the imbalanced host response in sepsis (5). Recently, sepsis has been defined as a dysregulated host response to infection, resulting in life-threatening damage to organs and tissues (6, 7). Timely antibiotics and systemic supportive care are the standard treatment options, but effective therapies for sepsis remain elusive (8), resulting in persistently high incidence and mortality rates.

Trillions of symbiotic bacteria colonize the human intestine and are mainly composed of *Bacillota* and *Bacteroidota* (9–11); these bacteria are also called the second genome and play a crucial role in maintaining human health (12). It is widely accepted that various diseases such as obesity and diabetes are caused by dysbiosis of the gut microbiota (13–15). Moreover, the gut microbiota affects host susceptibility and responsiveness to sepsis through multiple pathways (16), and microbial dysbiosis has been recognized as a remarkable contributor to increased susceptibility to sepsis and subsequent organ dysfunction (17–19). In recent years, some observational studies (19–25) have suggested that the gut microbiota is associated with the onset and progression of sepsis. However, in traditional observational studies, the association between the gut microbiota and sepsis has been shown to be influenced by confounding factors such as antibiotic use and dietary habits, as well as reverse causality, which limits the inference of causality. To investigate the causal effect between the gut microbiota and sepsis, large-sample and high-quality randomized controlled trials (RCTs) are still needed for further validation. However, because of objective factors such as technology, cost, and research methods, there are significant limitations in identifying the types of strains associated with early diagnosis and prognosis.

Mendelian randomization (MR) analysis is a novel approach for inferring causal associations that provides an alternative to RCTs. This method utilizes single-nucleotide polymorphisms (SNPs) identified by genome-wide association studies (GWASs) as instrumental variables (IVs) to explore the causal association between exposure (e.g., the abundance of the genus *Dialister*) and outcome (e.g., sepsis) (26). Mendel’s laws of inheritance dictate that parental alleles are randomly assigned to offspring, which is akin to random assignment in RCTs. Genetic variation, in theory, is not influenced by common confounding factors, such as the postnatal environment, and genetic variation precedes exposure and outcome, eliminating the issues of reverse causality and confounding factors. Large-scale GWAS data have provided a wealth of reliable genetic variation information for MR studies of the gut microbiota (26, 27), and many studies (28, 29) have utilized the two-sample MR method to investigate the causal associations between the gut microbiota and various diseases.

This study aimed to utilize summary statistics from the MiBioGen and UK Biobank consortiums and employ a two-sample MR approach to investigate the causal association between the gut microbiota and the onset and progression of sepsis.

## 2 Methods

### 2.1 Study design

**Figure 1:**
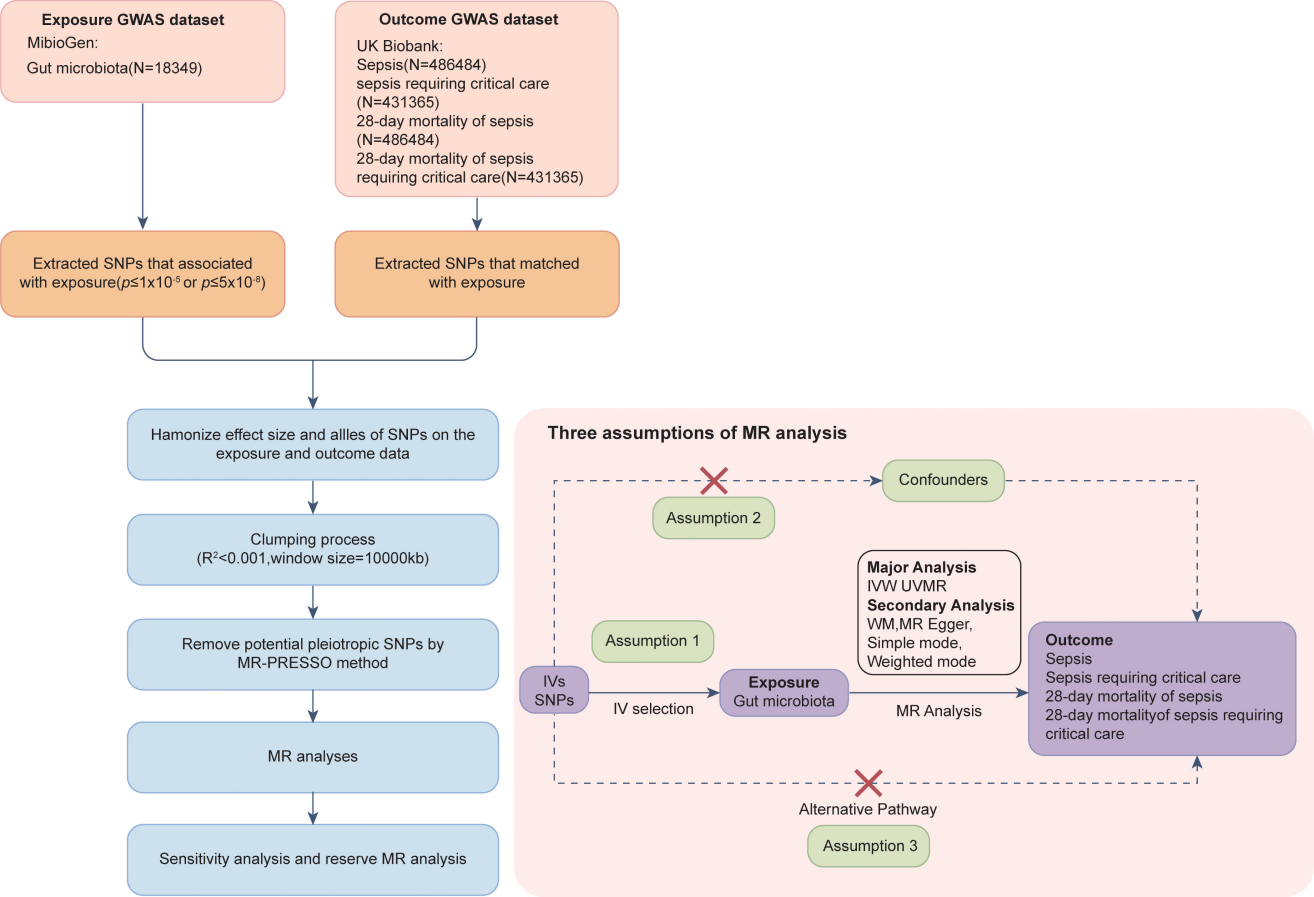
Synopsis of MR analysis procedures and major assumptions.

The flow chart of this MR analysis is shown in **Figure 1**. This study utilized publicly available GWAS summary statistics for a two-sample MR analysis to assess the causal association between the gut microbiota and the onset and progression of sepsis. Our MR analysis relied on three assumptions (26): (1) the IVs are strongly associated with the exposure; (2) the IVs are unrelated to confounding factors that affect the exposure–outcome association; and (3) the IVs only affect the outcome through the exposure and not through any other pathways. Moreover, this study was reported according to the Strengthening the Reporting of Observational Studies in Epidemiology Using Mendelian Randomization guidelines (STROBE-MR, S1 Checklist) (30).

### 2.2 Exposure GWAS datasets

The genetic variation in the gut microbiota in this study was derived from a genome-wide meta-analysis conducted by the MiBioGen consortium (31), which represents the largest gut microbiota GWAS to date. This study identified genetic associations between gut microbial relative abundances and human host genes. In this study, genotyping data and 16S ribosomal RNA gene sequencing profiles from 18,340 participants across 24 cohorts in Europe, America, the Middle East, and East Asia were coordinated. Twenty cohorts included samples of single ancestry, 16 of which were of European ancestry, for a total of 13,266 participants. The baseline characteristics of the exposure population can be viewed in **Supplementary Table S1**. This multiethnic large-scale GWAS divided the gut microbiota into 211 taxa (131 genera, 35 families, 20 orders, 16 classes, and 9 phyla). Fifteen bacterial taxa (12 genera and 3 families) with unknown groups were excluded, with 196 bacterial taxa finally included in our MR analysis. Summary-level GWAS data of the gut microbiota are openly available at http://www.mibiogen.org/.

### 2.3 Outcome GWAS datasets

Summary-level GWAS statistics of sepsis, sepsis requiring critical care, and 28-day mortality of patients with sepsis and sepsis requiring critical care were obtained from the UK Biobank consortium with adjustment for sex and age. The UK Biobank is a large and publicly available biomedical database and research resource. Since 2006, blood, urine, and saliva samples and complete demographic, socioeconomic, lifestyle, and health information data have been collected from approximately 500,000 participants aged 40 to 69 years throughout the United Kingdom (32). All the participants in the case and control groups (both men and women) included in the UK Biobank are of European descent. The phenotype “sepsis, sepsis requiring critical care, 28-day mortality of sepsis, and 28-day mortality of sepsis requiring critical care” was applied in our research. Comprehensive information on the diagnostic criteria and recruitment methods used for participants in the UK Biobank consortium can be found in the original publications. The profiles of the GWAS datasets of the gut microbiota and sepsis and sepsis-related outcomes are available in **Table 1**.

**Table 1:** Summary information of the datasets utilized in this MR analysis.

Trait	Consortium	Samples	Case	Control
Exposure				
Gut microbiota	MiBioGen	18,340	/	/
Outcome				
Sepsis	UK Biobank	486,484	11,643	474,841
Sepsis requiring critical care	UK Biobank	431,365	1,380	429,985
28-day mortality of sepsis	UK Biobank	486,484	1,896	484,588
28-day mortality of sepsis requiring critical care	UK Biobank	431,365	347	431,018

### 2.3 Instrumental variables

SNPs strongly associated with each bacterial trait were selected as IVs in our MR analysis. To ensure the reliability and accuracy of the results regarding the causal association between the gut microbiota and the risk of sepsis and sepsis-related outcomes, we utilized the following selection criteria to choose IVs: (1) To improve the completeness of our results, SNPs associated with each gut microbial taxon at the genome-wide significance threshold (*p* < 5×10**^−^** ^8^) and the locus-wide significance threshold (*p* < 1×10^–5^) were chosen as IVs (33). (2) Using the 1000 Genomes Project European sample data as the reference panel, this study conducted a clumping analysis (*r* ^2^ < 0.001, window size = 10,000 kilobases) to assess the linkage disequilibrium (LD) between the included SNPs and removed highly correlated SNPs to ensure that the included SNPs were independent of each other. (3) The exposure (gut microbiota) and outcome (sepsis and sepsis-related outcomes) data were harmonized, and palindromic SNPs with intermediate allele frequencies were removed. (4) The *F*-statistic for the IVs was calculated to evaluate potential bias due to weak IVs. An *F*-statistic > 10 was interpreted as an indication of negligible bias from weak IVs.

### 2.4 Statistical analysis

MR was conducted to analyze the causal relationships between the gut microbiota and sepsis and sepsis-related outcomes. The inverse-variance weighted (IVW) method was used as the primary method to identify potential causal associations, as it is regarded as the most powerful statistical method. A meta-analysis approach combined with the Wald estimates for each valid SNP was used to assess a total estimate of the effect of the exposure variables on outcome. For each bacterial trait of the gut microbiota, if the IVW method identified causality (*p* < 0.05), we performed the other four MR methods, MR−Egger, weighted median, simple mode, and weighted mode, to supplement the IVW results (34, 35). The MR−Egger method delivers unbiased estimates even when all chosen IVs exhibit pleiotropy, given that the Instrument Strength Independent of Direct Effect (InSIDE) assumption is satisfied (36). The weighted median method can still accurately estimate the causality effect even when less than 50% of the genetic variants violate the core assumptions of MR (34). Finally, we report the causal results as odds ratios (ORs) with 95% confidence intervals (95% CIs). The significance threshold was established at *p* < 0.05.

We considered an exposure–outcome pair to have a causal association only when all MR methods consistently identified the same direction of effect. To validate the robustness of the established causal associations, we conducted a series of sensitivity analyses. First, Cochran’s IVW *Q* statistics were calculated to quantify the heterogeneity. A *Q*-value exceeding the total number of IVs reduced by one suggested the presence of heterogeneity and potentially invalid IVs. Similarly, *Q* statistics that yielded a *p*-value < 0.05 also indicated the existence of heterogeneity (37, 38). Second, we performed MR−Egger analysis to assess the confounding effects of directional pleiotropy. When the intercept of the MR−Egger was close to zero at a *p*-value > 0.05, we regarded directional pleiotropy as not significant. Third, to assess overall pleiotropy, Mendelian randomization pleiotropy residual sum and outlier (MR-PRESSO) analysis was performed (39). We reported the outcomes of the MR-PRESSO global test, and outlier-corrected ORs and confidence intervals (CIs) were calculated for outliers and horizontal pleiotropic SNPs. Finally, to detect pleiotropy caused by a single SNP, a leave-one-out analysis was also performed.

To investigate whether sepsis and sepsis-related outcomes had any causal influence on the identified significant gut microbiota, we also conducted reverse-direction MR analysis on bacteria with significant causal associations in forward-direction MR. The settings and methods were identical to those used for forward-direction MR.

All the statistical analyses were performed using R version 4.2.3 (R Foundation for Statistical Computing, Vienna, Austria, https://www.r-project.org/). MR analyses were performed using TwosampleMR (version 0.5.6) (26) and MR-PRESSO (version 1.0) (39).

## 3 Results

**Figure 2:**
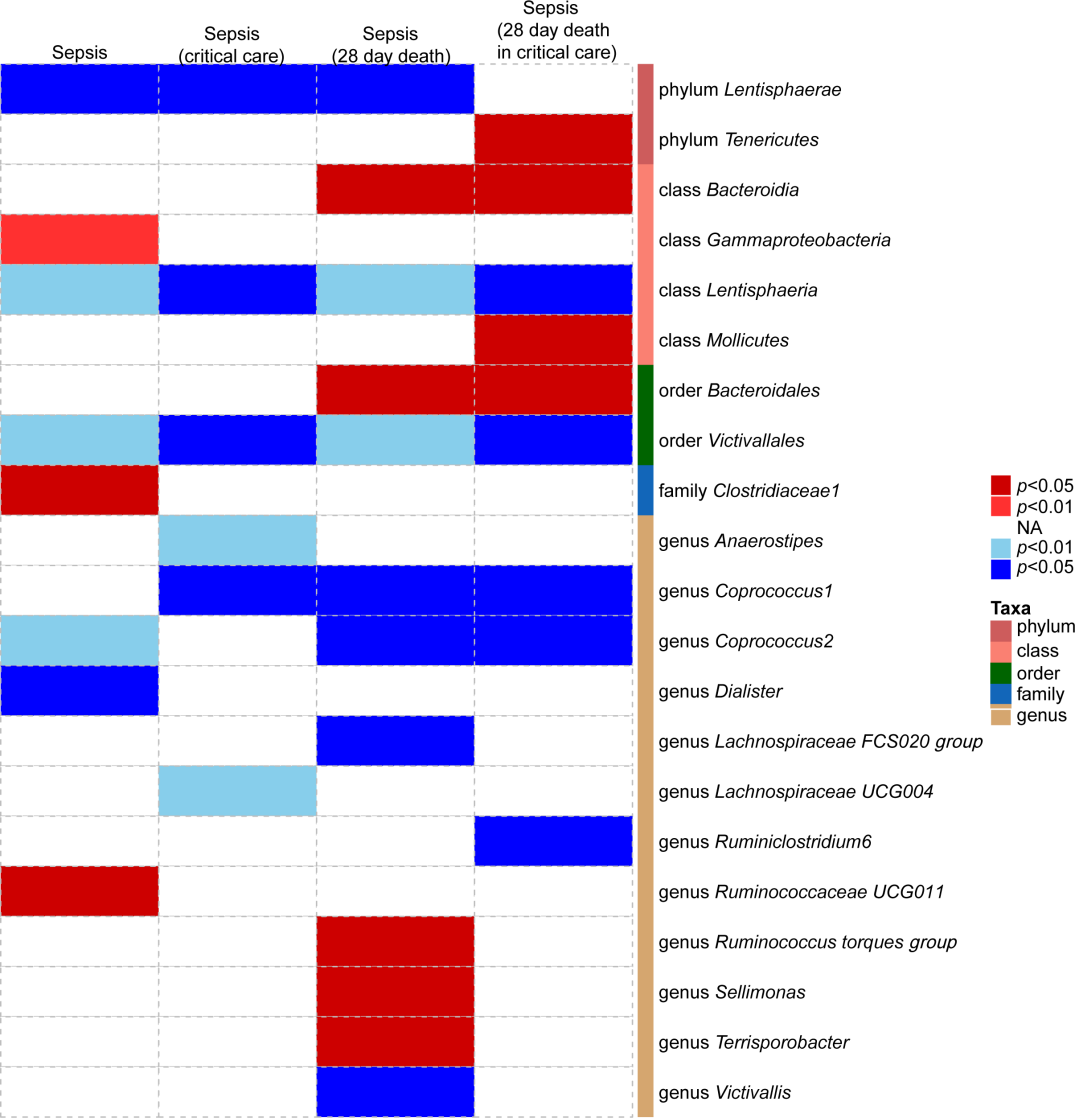
Heatmap of gut microbiota causally associated with sepsis, sepsis requiring critical care, 28-day mortality of sepsis, and 28-day mortality of sepsis requiring critical care identified by the IVW method. Red represents risk factors, whereas blue represents protective factors.

The details of the selected SNPs are shown in **Supplementary Table S2** (i.e., SNPID, effect allele, other allele, beta, standard error, and *p*-value of exposure and outcome). Based on the selection criteria for IVs, we identified 196 traits of the gut microbiota at five biological levels (i.e., phylum, class, order, family, and genus) associated with sepsis and sepsis-related outcomes (**Supplementary Table S3**). As shown in **Table 2**, 8, 6, 12, and 9 bacterial traits were potentially causally associated with sepsis, sepsis requiring critical care, 28-day mortality from sepsis, and 28-day mortality from sepsis requiring critical care, respectively, according to the IVW MR analysis. Following the harmonization process, every pair of bacterial traits and sepsis and sepsis-related outcomes incorporated more than three SNPs. All of the *F*-statistics of the selected IVs in this research were greater than 10, suggesting that there was no weak instrument bias. It is important to acknowledge that the classifications of the gut microbiota have a considerable degree of overlap. Consequently, the SNPs included in the class and their corresponding order could coincide significantly (e.g., SNPs of the phylum *Lentisphaerae*, class *Lentisphaeria*, order *Victivallales*, and genus *Victivallis*). A heatmap was generated to visualize the causal association of bacterial traits identified in our MR analysis with sepsis, sepsis requiring critical care, and 28-day mortality of sepsis and sepsis requiring critical care (**Figure 2**).

**Table 2:** MR results of causal effects between gut microbiota and sepsis and sepsis-related outcomes (*p* < 1×10^−5^).

Gut microbiota (exposure)	Method	nSNP	OR	95% CI	*p-*value	Egger intercept	Egger_intercept *p-*value	Cochrane *Q* statistic	Cochrane *Q p-*value	MR-PRESSO
Sepsis										
Class *Gammaproteobacteria*	IVW	6	1.37	1.08–1.73	0.010	−0.0009	0.979	7.1272	0.211	/
Class *Lentisphaeria*	IVW	8	0.86	0.78–0.94	0.002	0.0125	0.628	5.1588	0.641	/
Family *Clostridiaceae1*	IVW	10	1.21	1.04–1.40	0.011	−0.0242	0.168	5.6311	0.776	/
Genus *Coprococcus2*	IVW	8	0.81	0.69–0.94	0.007	0.0217	0.645	4.1443	0.763	/
Genus *Dialister*	IVW	11	0.85	0.74–0.97	0.016	−0.0092	0.658	4.7209	0.909	/
Genus *Ruminococcaceae UCG011*	IVW	8	1.10	1.01–1.20	0.024	−0.0036	0.909	6.1743	0.520	/
Order *Victivallales*	IVW	8	0.86	0.78–0.94	0.002	0.0125	0.628	5.1588	0.641	/
Phylum *Lentisphaerae*	IVW	9	0.89	0.80–0.99	0.035	0.0091	0.781	11.3614	0.182	/
Sepsis (critical care)										
Class *Lentisphaeria*	IVW	8	0.67	0.50–0.91	0.011	−0.0387	0.662	8.8974	0.260	/
Genus *Anaerostipes*	IVW	11	0.49	0.31–0.76	0.002	0.0328	0.507	7.7479	0.653	/
Genus *Coprococcus1*	IVW	11	0.65	0.43–1.00	0.049	0.0100	0.807	11.6969	0.306	/
Genus *Lachnospiraceae UCG004*	IVW	12	0.51	0.34–0.77	0.001	0.0406	0.467	11.9762	0.365	/
Order *Victivallales*	IVW	8	0.67	0.50–0.91	0.011	−0.0387	0.662	8.8974	0.260	/
Phylum *Lentisphaerae*	IVW	9	0.70	0.53–0.93	0.014	−0.0443	0.610	9.7340	0.284	/
Sepsis (28-day death)										
Class *Bacteroidia*	IVW	13	1.48	1.06–2.08	0.023	0.0187	0.533	7.9222	0.791	/
Class *Lentisphaeria*	IVW	8	0.68	0.53–0.87	0.002	−0.0070	0.921	7.7980	0.351	/
Genus *Coprococcus1*	IVW	11	0.67	0.48–0.94	0.020	0.0233	0.450	7.0174	0.724	/
Genus *Coprococcus2*	IVW	8	0.48	0.27–0.86	0.013	−0.0129	0.945	15.9305	0.026	/
Genus *Lachnospiraceae FCS020 group*	IVW	12	0.70	0.52–0.95	0.023	0.0395	0.202	8.8919	0.632	/
Genus *Ruminococcus torques group*	IVW	8	1.53	1.00–2.35	0.049	−0.0656	0.118	5.9762	0.543	/
Genus *Sellimonas*	IVW	9	1.25	1.04–1.50	0.019	0.0149	0.850	6.3185	0.612	/
Genus *Terrisporobacter*	IVW	5	1.43	1.02–2.02	0.040	0.0376	0.513	2.7535	0.600	/
Genus *Victivallis*	IVW	9	0.82	0.68–0.99	0.042	−0.0003	0.998	2.3065	0.970	/
Order *Bacteroidales*	IVW	13	1.48	1.06–2.08	0.023	0.0187	0.533	7.9222	0.791	/
Order *Victivallales*	IVW	8	0.68	0.53–0.87	0.002	−0.0070	0.921	7.7980	0.351	/
Phylum *Lentisphaerae*	IVW	9	0.72	0.56–0.93	0.012	−0.0131	0.866	10.7141	0.218	/
Sepsis (28-day death in critical care)										
Class *Bacteroidia*	IVW	13	2.43	1.10–5.37	0.029	−0.0163	0.817	7.8873	0.794	/
Class *Lentisphaeria*	IVW	8	0.54	0.30–0.95	0.034	0.0031	0.985	7.6435	0.365	/
Class *Mollicutes*	IVW	12	2.03	1.01–4.08	0.046	0.0251	0.809	7.0423	0.796	/
Genus *Coprococcus1*	IVW	11	0.42	0.19–0.92	0.031	−0.0236	0.743	2.0631	0.996	/
Genus *Coprococcus2*	IVW	8	0.34	0.14–0.83	0.018	−0.0311	0.908	3.6194	0.822	/
Genus *Ruminiclostridium6*	IVW	14	0.43	0.22–0.83	0.012	0.1002	0.197	12.9286	0.453	/
Order *Bacteroidales*	IVW	13	2.43	1.10–5.37	0.029	−0.0163	0.817	7.8873	0.794	/
Order *Victivallales*	IVW	8	0.54	0.30–0.95	0.034	0.0031	0.985	7.6435	0.365	/
Phylum *Tenericutes*	IVW	12	2.03	1.01–4.08	0.046	0.0251	0.809	7.0423	0.796	/

IVW, inverse-variance weighted method; nSNP, number of the SNP used as the IVs for the MR analyses; OR, odds ratio; CI, confidence interval.

### 3.1 MR analysis results (locus-wide significance, *p* < 1×10^−5^)

#### 3.1.1 Causality of the gut microbiota on sepsis

**Figure 3:**
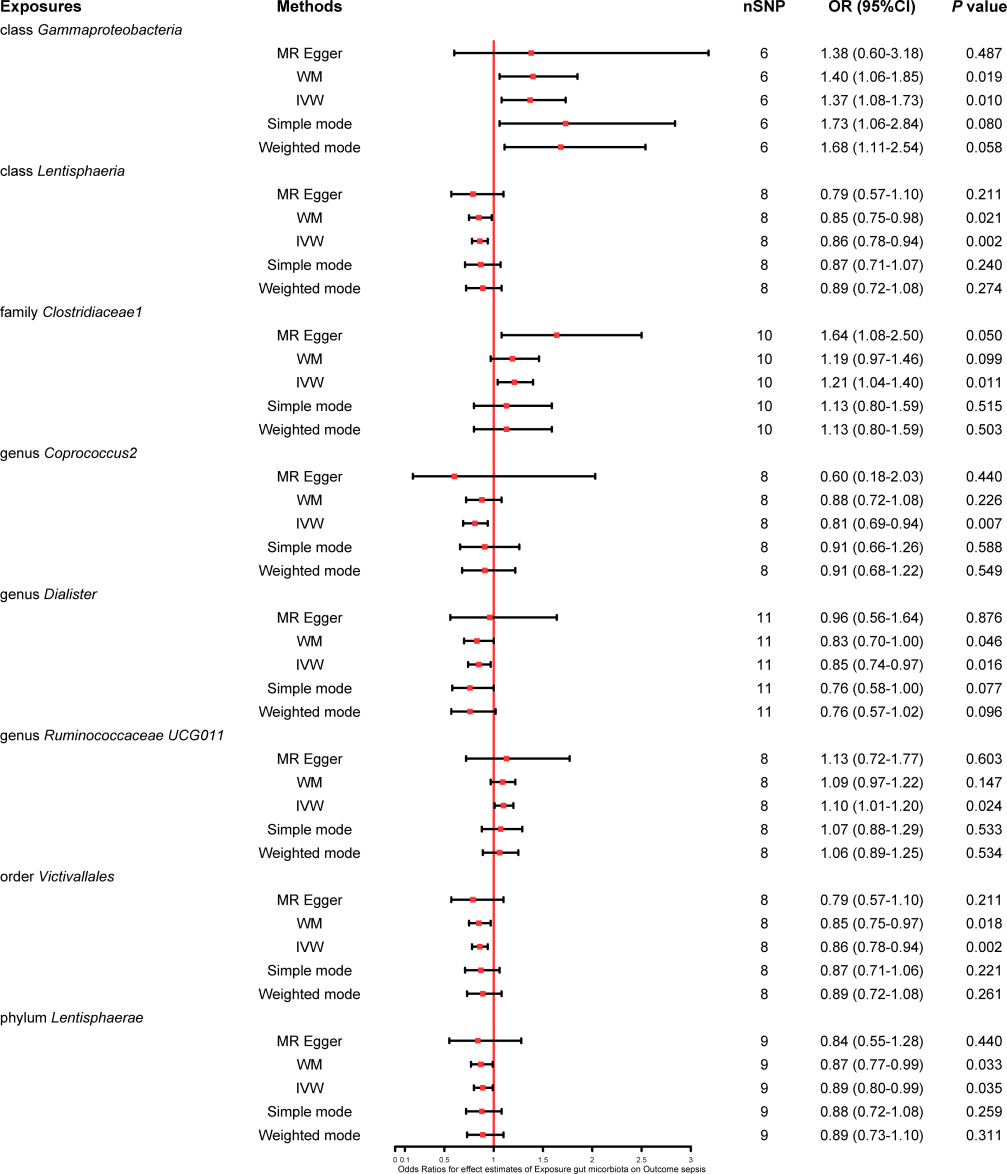
Forest plot of various MR results for eight bacterial traits causally associated with sepsis.

We found that five bacterial traits (class *Lentisphaeria*: OR 0.86, 95% CI 0.78–0.94; genus *Coprococcus2*: OR 0.81, 95% CI 0.69–0.94; genus *Dialister*: OR 0.85, 95% CI 0.74–0.97; order *Victivallales*: OR 0.86, 95% CI 0.78–0.94; and phylum *Lentisphaerae*: OR 0.89, 95% CI 0.80–0.99) had a potential protective effect on sepsis, while three bacterial traits (class *Gammaproteobacteria*: OR 1.37, 95% CI 1.08–1.73; family *Clostridiaceae1*: OR 1.21, 95% CI 1.04–1.40; and genus *Ruminococcaceae UCG011*: OR 1.10, 95% CI 1.01–1.20) were causally associated with a greater risk of sepsis according to the IVW MR analysis. However, the weighted mode method revealed that five bacterial traits had a significant causal association with the risk of sepsis (class *Gammaproteobacteria*: OR 1.40, 95% CI 1.06–1.85; class *Lentisphaeria*: OR 0.85, 95% CI 0.75–0.98; order *Victivallales*: OR 0.85, 95% CI 0.75–0.97; phylum *Lentisphaerae*: OR 0.87, 95% CI 0.77–0.99; and genus *Dialister*: OR 0.83, 95% CI 0.70–1.00). Moreover, the results of MR−Egger regression showed that only the family *Clostridiaceae1* (OR 1.64, 95% CI 1.08–2.50) was significantly associated with the risk of sepsis. The comprehensive MR results of the causal associations between bacterial traits and sepsis are shown in **Figure 3**.

#### 3.1.2 Causality of the gut microbiota on sepsis requiring critical care

**Figure 4:**
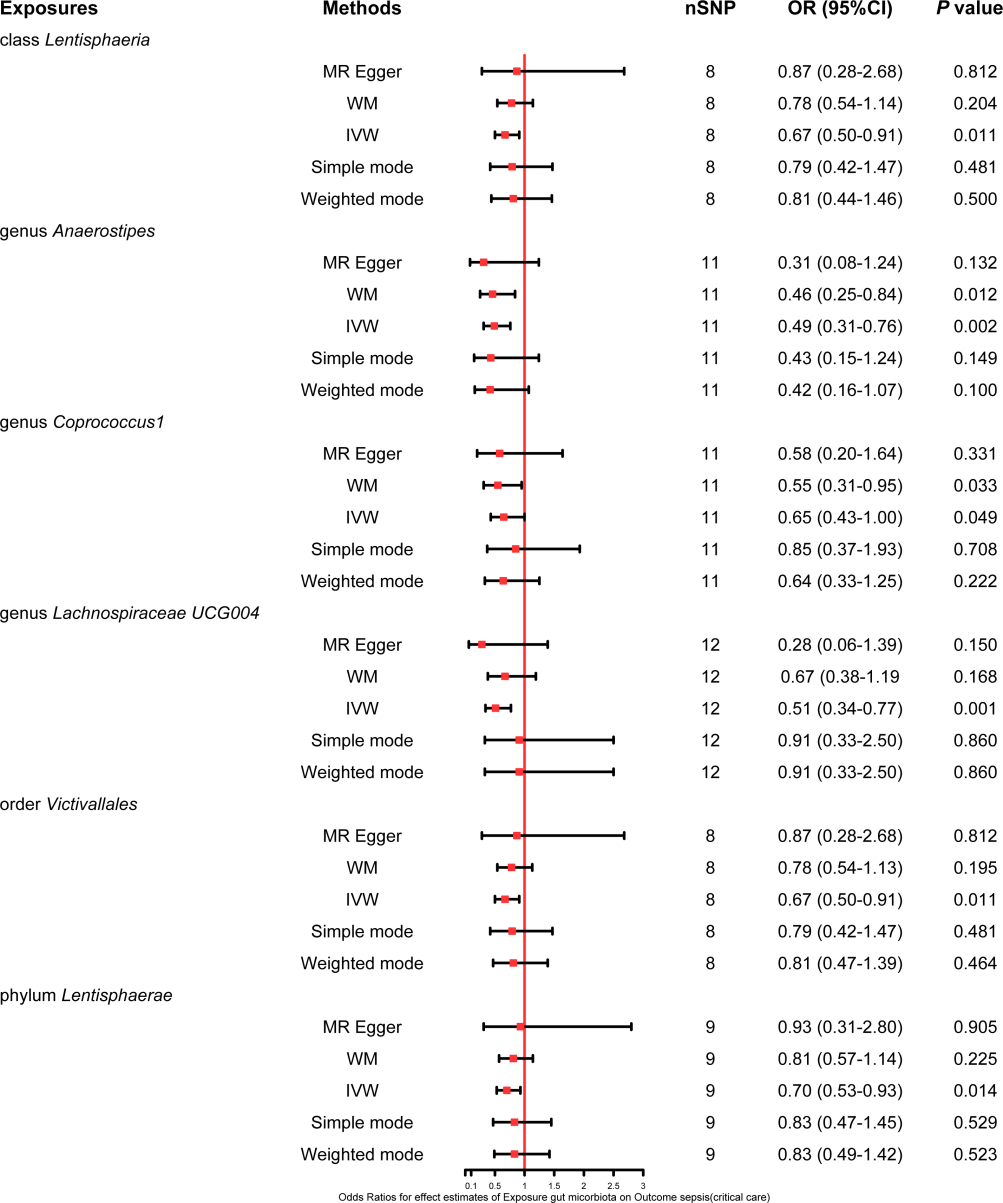
Forest plot of various MR results for six bacterial traits causally associated with sepsis requiring critical care.

This study also explored the causal effect of the gut microbiota on the risk of sepsis requiring critical care. All six bacterial traits (class *Lentisphaeria*: OR 0.67, 95% CI 0.50–0.91; genus *Anaerostipes*: OR 0.49, 95% CI 0.31–0.76; genus *Coprococcus1*: OR 0.65, 95% CI 0.43–1.00; genus *Lachnospiraceae UCG004*: OR 0.51, 95% CI 0.34–0.77; order *Victivallales*: OR 0.67, 95% CI 0.50–0.91; and phylum *Lentisphaerae*: OR 0.70, 95% CI 0.53–0.93) were significantly associated with a potential protective effect on sepsis requiring critical care in the primary IVW MR analysis. Moreover, the results of the weighted mode method demonstrated that the genus *Anaerostipes* (OR 0.46, 95% CI 0.25–0.84) and *Coprococcus1* (OR 0.55, 95% CI 0.31–0.95) were also associated with a lower risk of sepsis requiring critical care. The comprehensive MR results concerning the causal association between bacterial traits and sepsis requiring critical care are depicted in **Figure 4**.

#### 3.1.3 Causality of the gut microbiota on 28-day mortality from sepsis

**Figure 5:**
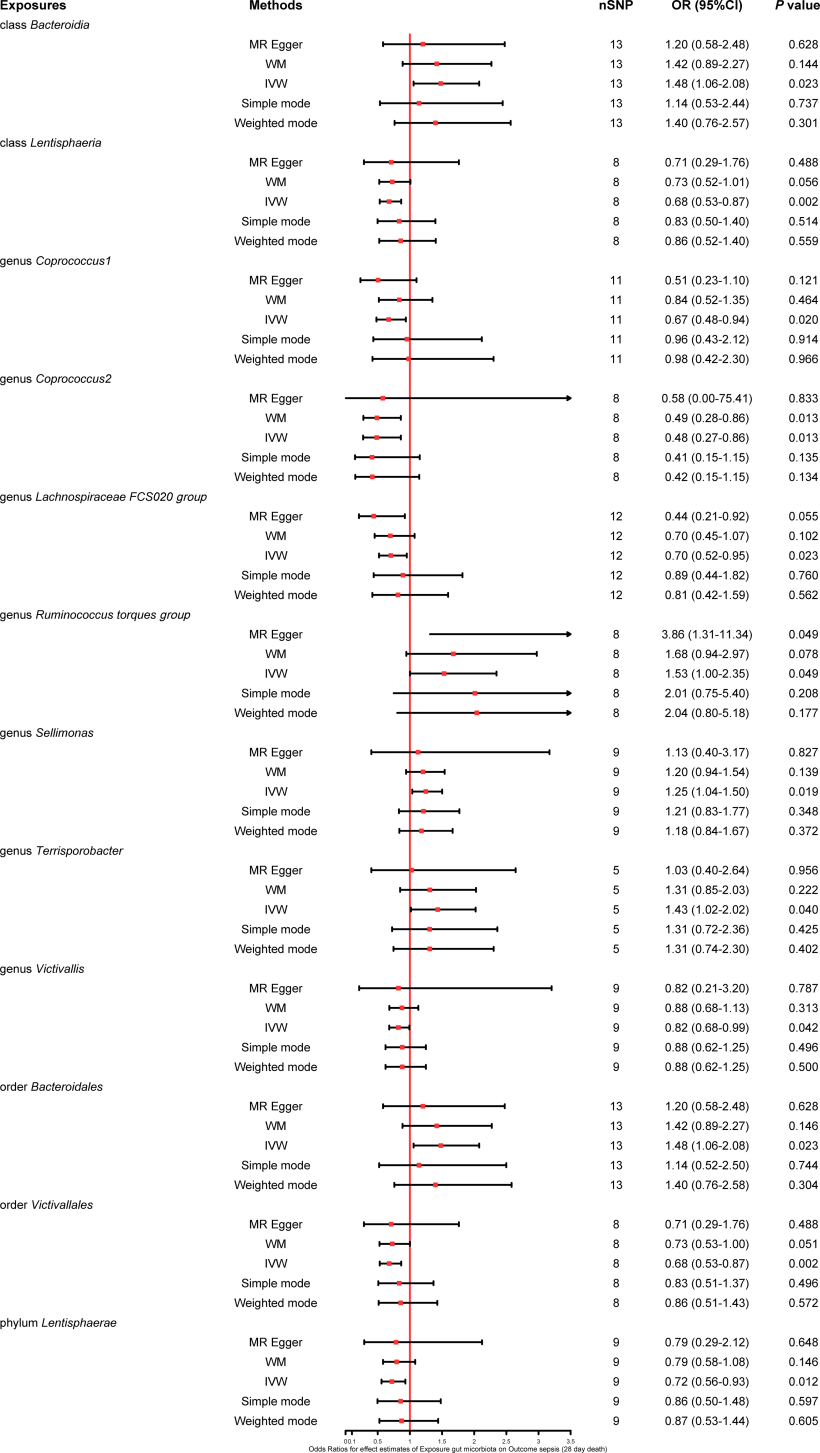
Forest plot of various MR results for 12 bacterial traits causally associated with 28-day mortality of sepsis.

Five bacterial traits (class *Bacteroidia*: OR 1.48, 95% CI 1.06–2.08; genus *Ruminococcus torques group*: OR 1.53, 95% CI 1.00–2.35; genus *Sellimonas*: OR 1.25, 95% CI 1.04–1.50; genus *Terrisporobacter*: OR 1.43, 95% CI 1.02–2.02; and order *Bacteroidales*: OR 1.48, 95% CI 1.06–2.08) were significantly associated with an increase in 28-day mortality from sepsis, while seven other bacterial traits (class *Lentisphaeria*: OR 0.68, 95% CI 0.53–0.87; genus *Coprococcus1*: OR 0.67, 95% CI 0.48–0.94; genus *Coprococcus2*: OR 0.48, 95% CI 0.27–0.86; genus *Lachnospiraceae FCS020 group*: OR 0.70, 95% CI 0.52–0.95; genus *Victivallis*: OR 0.82, 95% CI 0.68–0.99; order *Victivallales*: OR 0.68, 95% CI 0.53–0.87; and phylum *Lentisphaerae*: OR 0.72, 95% CI 0.56–0.93) were reported to be significantly associated with a lower risk of 28-day mortality from sepsis in the primary IVW MR analysis. Moreover, the weighted median method showed that the genus *Coprococcus2* had a significant protective effect on 28-day mortality from sepsis (OR 0.49, 95% CI 0.28–0.86), and the MR−Egger regression showed that the genus *Ruminococcus torques group* (OR 3.86, 95% CI 1.31–11.34) was associated with a greater risk of 28-day mortality from sepsis. The detailed results from the MR analysis showing the causal relationships between bacterial traits and 28-day mortality from sepsis are illustrated in **Figure 5**.

#### 3.1.4 Causality of the gut microbiota on 28-day mortality from sepsis requiring critical care

**Figure 6:**
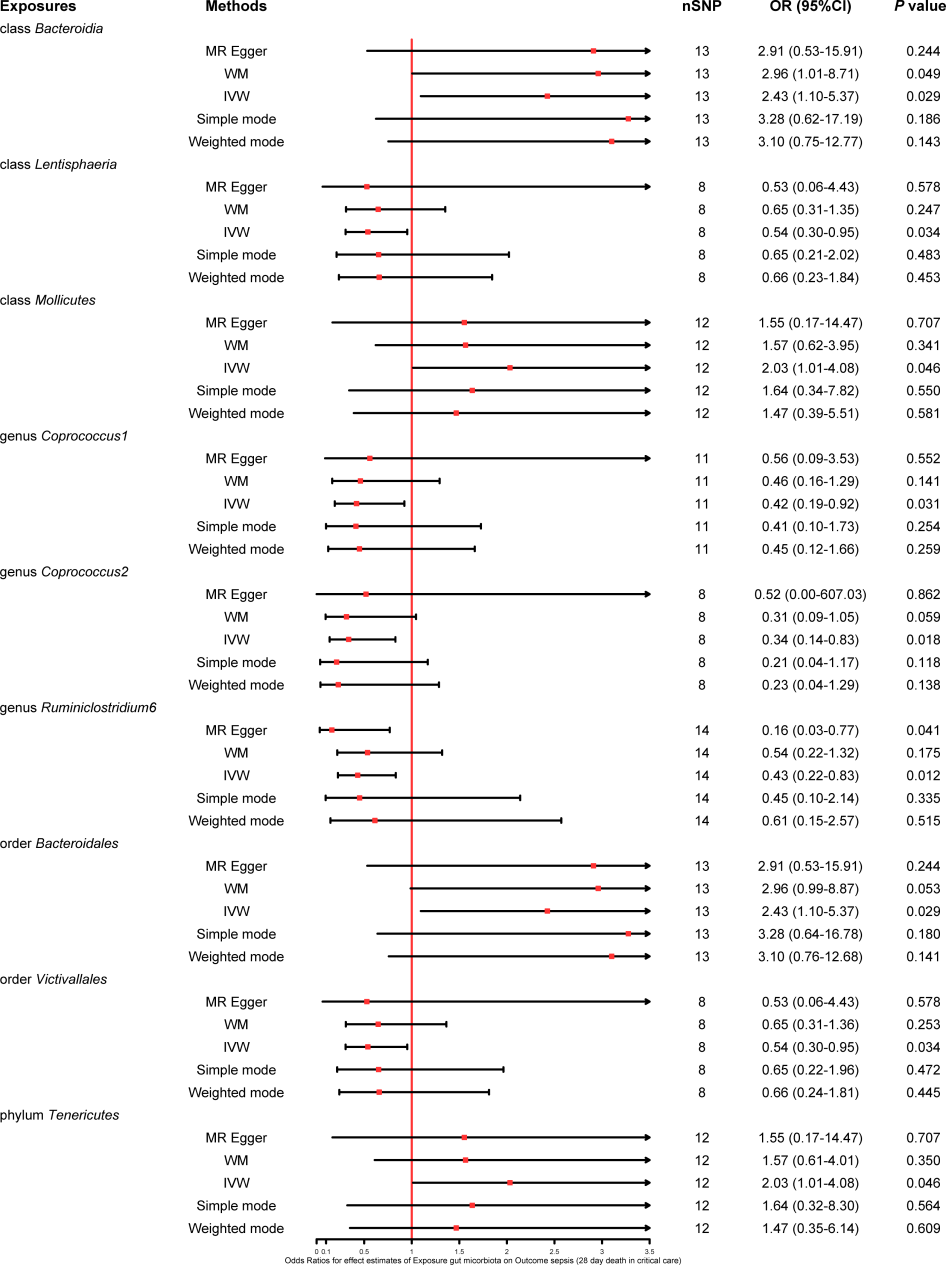
Forest plot of various MR results for nine bacterial traits causally associated with 28-day mortality of sepsis requiring critical care.

The results of the primary IVW MR analysis showed that four bacterial traits (class *Bacteroidia*: OR 2.43, 95% CI 1.10–5.37; class *Mollicutes*: OR 2.03, 95% CI 1.01–4.08; order *Bacteroidales*: OR 2.43, 95% CI 1.10–5.37; and phylum *Tenericutes*: OR 2.03, 95% CI 1.01–4.08) were significantly associated with a greater risk of 28-day mortality from sepsis requiring critical care, and five bacterial traits (class *Lentisphaeria*: OR 0.54, 95% CI 0.30–0.95; genus *Coprococcus1*: OR 0.42, 95% CI 0.19–0.92; genus *Ruminiclostridium6*: OR 0.43, 95% CI 0.22–0.83; genus *Coprococcus2*: OR 0.34, 95% CI 0.14–0.83; and order *Victivallales*: OR 0.54, 95% CI 0.30–0.95) were causally associated with a lower risk of 28-day mortality from sepsis requiring critical care, suggesting a potential protective effect. Moreover, the estimates of the weighted median method showed that the class *Bacteroidia* was significantly associated with 28-day mortality from sepsis requiring critical care (OR 2.96, 95% CI 1.01–8.71). MR−Egger regression revealed that the genus *Ruminiclostridium6* was significantly associated with 28-day mortality from sepsis requiring critical care (OR 0.16, 95% CI 0.03–0.77). The extensive MR findings on the potential causal link between bacterial traits and 28-day mortality from sepsis requiring critical care are displayed in **Figure 6**.

#### 3.1.5 Sensitivity analysis

**Figure 7:**
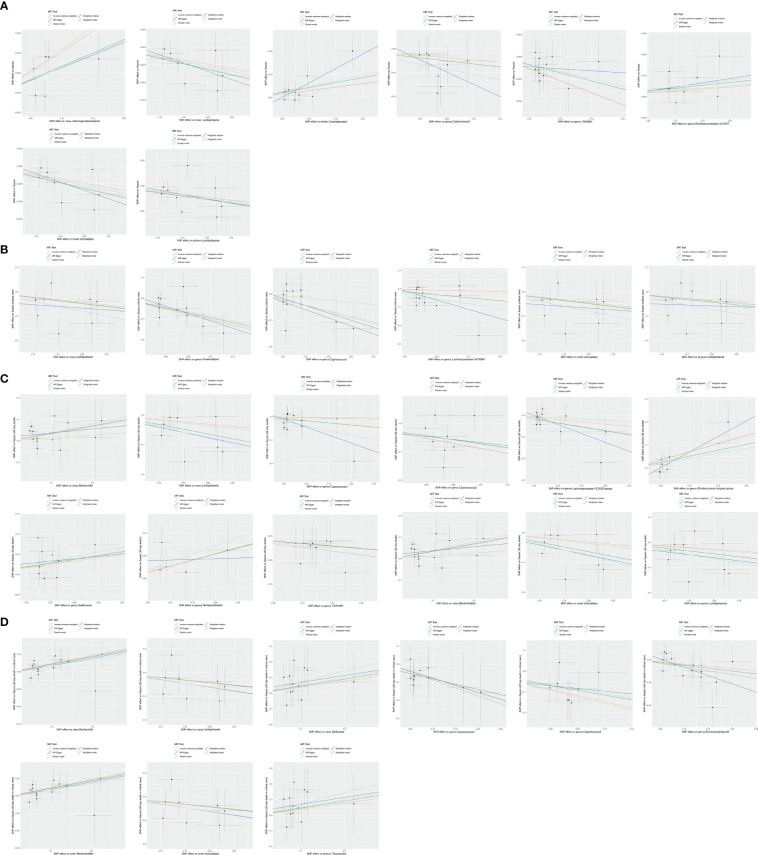
Scatter plot of MR results. **(A)** Scatter plot of genetic correlations of eight bacterial traits and sepsis using five MR methods. **(B)** Scatter plot of genetic correlations of six bacterial traits and sepsis requiring critical care using five MR methods. **(C)** Scatter plot of genetic correlations of 12 bacterial traits and 28-day mortality of sepsis using five MR methods. **(D)** scatter plot of genetic correlations of nine bacterial traits and 28-day mortality of sepsis requiring critical care using five MR methods.

**Figure 8:**
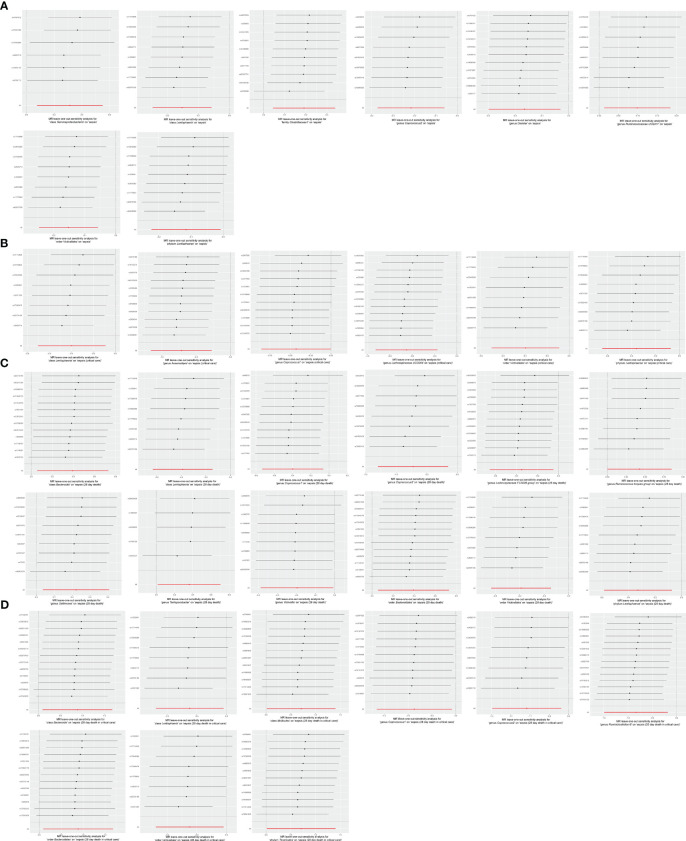
Leave-one-out analysis for **(A)** 8 bacterial traits on sepsis, **(B)** 6 bacterial traits on sepsis requiring critical care, **(C)** 12 bacterial traits on 28-day mortality of sepsis, and **(D)** 9 bacterial traits on 28-day mortality of sepsis requiring critical care.

The robustness of the MR analysis results was confirmed by scatter plots (**Figures 7A–D**) and leave-one-out plots (**Figures 8A–D**). According to the MR−Egger regression intercept methods, there was no evidence of horizontal pleiotropy for these 21 bacterial traits, with causal associations with sepsis and sepsis-related outcomes (**Supplementary Table S4**). Potentially significant heterogeneity was detected only for the association between 28-day mortality from sepsis and the genus *Coprococcus2* (Cochran’s *Q* statistics = 15.93, *p* = 0.026) (**Supplementary Table S4**). Moreover, we found no significant heterogeneity (*p* > 0.05) according to Cochran’s IVW *Q* statistics of the remaining 20 bacterial traits. Visual examination clearly revealed that the removal of any single IV did not significantly affect the overall results. Furthermore, MR-PRESSO tests showed the absence of outliers in the results (**Supplementary Table S5**).

### 3.2 Results of the MR analysis (locus-wide significance, *p* < 5×10^−8^)

In the MR analysis of the gut microbiota and its relationship with sepsis and sepsis-related outcomes, none of the five MR methods identified any significant causal associations. When a sensitivity analysis was conducted, no evidence of heterogeneity was found according to Cochrane’s *Q* test. Furthermore, no horizontal pleiotropy was detected by either the MR−Egger intercept test or the MR-PRESSO global test, and no outliers were identified by the MR-PRESSO outlier test. The full results can be found in **Supplementary Table S6**.

### 3.3 Reverse-direction MR analyses

The reverse MR analysis results suggested that there is no causal effect of septic traits on bacterial traits (**Supplementary Table S7**).

## 4 Discussion

To our knowledge, this is the first MR analysis to comprehensively explore the causal effect of the gut microbiota on sepsis onset, progression, and mortality using publicly available genetic databases. In this study, MR analyses were performed on 196 bacterial traits to reveal the potential role of the gut microbiota in the onset and progression of sepsis. We found that 21 causal bacterial traits have a critical impact on the onset and progression of sepsis. Notably, two bacterial traits of the gut microbiota (*Victivallales* and *Lentisphaeria*) are the same, and therefore, we only report the results for *Victivallales*.

Muratsu et al. (40) noted an increase in *Ruminococcaceae* abundance during the subacute phase of sepsis in mice, suggesting a potential association between the presence of *Ruminococcaceae* and sepsis. However, Stoma et al. (41) reported a negative association between *Ruminococcaceae* and sepsis risk in a population study, which contrasts with our findings. Moreover, Zhang et al. (42) reported that the presence of *Ruminococcaceae* in rats was negatively associated with lipopolysaccharide (LPS)-binding protein (LBP) and proinflammatory factors, such as interleukin-6 (IL-6) and tumor necrosis factor-α (TNF-α). Our research, for the first time, suggested that *Ruminococcaceae* may play a role in causing sepsis, potentially serving as a novel biomarker. Based on our findings, we propose that the effects of *Ruminococcaceae* on sepsis may depend on the specific species and strains. Burritt et al. (43) reported that the presence of *Gammaproteobacteria* in rats subjected to cecal ligation and puncture was positively associated with sepsis risk. *Gammaproteobacteria* has been shown to be positively associated with the pathways of severe LPS-related hyperinflammatory stress, which is a risk factor for sepsis in patients with decompensated cirrhosis (44). Based on our results, as with *Proteobacteria*, *Gammaproteobacteria* are considered to have proinflammatory properties (45–47), which increase the risk of sepsis. A study conducted by Arimatsu et al. (48) revealed that after mice were exposed to oral pathogens belonging to *Bacteroidales*, a significant positive association was observed between *Bacteroidales* and systemic inflammation. Furthermore, consistent with our findings, a positive association between *Bacteroidales* and the proinflammatory cytokine TNF-α was identified (48), with TNF-α known to be associated with the progression of sepsis (49). Consistent with our results, *Lachnospiraceae* has been shown to have health-promoting functions (50) and to play important roles in ulcerative colitis, diabetes, the immune response, and nutrient metabolism (51–55). Similarly, Peng et al. (56) reported a negative association between *Lachnospiraceae* and sepsis in the small intestines of mice. Moreover, Yu et al. (57) reported that *Lachnospiraceae* in septic mice fed a methyl diet was negatively associated with mortality, organ injury, and circulating levels of inflammatory mediators. Furthermore, Gai et al. (58) reported that the abundance of *Lachnospiraceae* in mice in the fecal microbiota transplantation (FMT) group was considerably greater than that in the control group, while septic mice in the FMT group exhibited reduced morbidity and mortality. There is a close relationship between the gut microbiota and the immune system (5, 59). IL-6 is a crucial cytokine involved in the innate immune response in sepsis, contributing to adverse outcomes in tandem with other pathophysiological processes (60–63). Moreover, animal models have shown that the elimination of proinflammatory cytokines such as TNF-α, IL-1b, IL-12, and IL-18 provides substantial protection against organ damage and mortality (49). There is now a consensus that the uncontrolled activity of proinflammatory cytokines contributes to sepsis-related injury.

Short-chain fatty acids (SCFAs), which primarily consist of acetic acid, propionic acid, and butyric acid, are the main end products of gut microbiota metabolism in the human body. This study identified a subset of the gut microbiota associated with the onset and progression of sepsis, which included SCFA-producing bacteria such as *Coprococcus* (64), *Dialister* (65), *Lachnospiraceae* (66), *Anaerostipes* (67), *Ruminococcaceae* (66), *Ruminococcus* (68), and *Ruminiclostridium* (69). Clinical and animal studies have shown that gut-derived SCFAs are associated with decreased sepsis risk and organ protection in patients with sepsis (70, 71). *Coprococcus*, an SCFA-producing bacteria, was reported to decrease in patients with sepsis (72, 73), suggesting a negative association between *Coprococcus* and the risk of sepsis. Furthermore, previous mice experiments revealed that the presence of *Coprococcus* in septic mice pretreated with *Lactobacillus rhamnosus* GG was negatively associated with mortality (74, 75). Based on our findings, *Coprococcus* could be a protective factor against sepsis, suggesting a possible mechanism by which *Coprococcus* regulates the progression of sepsis by producing SCFAs. Furthermore, consistent with our findings, *Lachnospiraceae* has been shown to have the greatest contribution to intestinal protection through L-lysine fermentation to SCFAs, such as acetate and butyrate (76, 77). These substances play critical roles in maintaining immune balance and suppressing inflammation (78–80), thereby enhancing the preventative and therapeutic efficacy against sepsis. Similarly, consistent with our results, *Ruminiclostridium*, a butyrate-producing bacteria, has been shown to be negatively associated with the proportion of inflammatory factors (81), which might reduce the inflammatory reaction and severity in sepsis.

Dysbiosis of the gut microbiota (an increase in pathogenic bacteria) may be a cause of bacterial sepsis (82). In the presence of a protective commensal microbiota, pathogenic bacteria in the gut of healthy hosts may not proliferate or cause disease, but the absence of a protective microbiota can lead to an overgrowth of pathogenic bacteria (83, 84). In a study by Hyoju et al. (85), mice were fed a high-fat or normal-fat diet, given broad-spectrum antibiotics, and then underwent partial hepatectomy. Compared to mice fed a normal diet, mice fed a high-fat diet had reduced microbial diversity in their gut microbiota, lower postoperative survival rates, an increase in multidrug-resistant Gram-negative bacteria, more intestinal bacterial spread, and higher mortality rates. Moreover, some large-scale observational studies on patients have provided indirect evidence that disruption of the gut microbiota is likely to cause sepsis (18, 20, 21). Features of the gut microbiota in individuals with sepsis include diminished diversity; decreased relative abundance of taxa such as *Bacillota* and *Bacteroidetes*; decreased numbers of symbiotic bacteria such as *Faecalibacterium*, *Blautia*, and *Ruminococcus*; and excessive growth of potential pathogens, including *Enterobacter*, *Enterococcus*, and *Staphylococcus* (86–88). Similarly, significant alterations in the microbiota may be linked to the progression of sepsis (89). Research indicates that the gut microbiota plays a role and is a major risk factor for late-onset sepsis (90, 91). Furthermore, Du et al. (22) discovered that an imbalance in the gut microbiota is associated with increased mortality rates and that the gut microbiota can serve as a prognostic indicator for sepsis.

Maintaining a fine equilibrium between harmful pathogens and beneficial probiotics in the gut is crucial for preserving the function of the intestinal barrier (92). Hyoju et al. (85) reported that compared to mice fed a regular diet, mice fed a high-fat diet had decreased α-diversity of the gut microbiota, increased mortality rates, and more gut microbiota taxa from the intestine that spread throughout the body. Moreover, impairment of intestinal barrier function can increase the entry of LPS produced by the gut microbiota into the blood (92), triggering systemic inflammation. This reduces the host’s ability to defend against infections, which may increase the risk of sepsis or further exacerbate immune dysregulation, ultimately leading to multiple organ failure. Some probiotics (such as *Lachnospiraceae*) have been proven to exhibit negative associations with intestinal permeability and plasma LPS levels (93). Furthermore, some SCFAs produced by probiotics, such as butyrate, are the main energy sources for intestinal epithelial cells. They participate in cell proliferation and differentiation, maintaining cellular homeostasis through anti-inflammatory and antioxidant effects (94, 95). In addition, SCFAs can influence the function of epithelial cells (70). Butyrate is known for both strengthening intestinal epithelial health and reinforcing barrier function (96) and is key for protecting against antigens such as endotoxins. Acetate shields mice from intestinal *Escherichia coli* translocation by influencing epithelial cell functions (97).

A leaky gut could be a cause or consequence of bacterial sepsis. Severe defects in the gut barrier can lead to the translocation of viable bacteria and bacteremia. This was shown in a study where mice with a leaky gut caused by dextran sulfate solution had higher levels of bacterial DNA in their blood (98). On the other hand, during sepsis, damage to the epithelial tight junctions in the intestines can contribute to the development of a leaky gut (99). In both scenarios, a leaky gut amplifies systemic inflammation through innate immune responses, particularly involving macrophages and neutrophils (100–102), which causes the onset and exacerbation of sepsis. *Bacteroidales* is significantly positively associated with the levels of endotoxin in the blood and significantly negatively associated with the gene expression of ileal tight junction proteins (48). Based on our results, we speculate that dysbiosis of the gut microbiota could enhance the translocation of *Bacteroidales* by increasing intestinal permeability and impairing the mucosal immune function of the gut, thereby exacerbating sepsis. Moreover, Palmieri et al. (103) reported that *Ruminococcus torques* degrades gastrointestinal mucin in patients with Crohn’s disease, impairing the mucus barrier produced by intestinal epithelial cells (IECs). The mucus barrier separates intestinal immune cells from the microbial community, reducing intestinal permeability. Impaired intestinal permeability and mucosal immune function can lead to the translocation of pathogenic microorganisms, triggering the excessive production of inflammatory factors and ultimately causing or worsening sepsis (104). However, consistent with our results, *Lachnospiraceae* exhibits a protective effect, which has a negative association with intestinal permeability and plasma LPS levels (93) and prevents the excessive transfer of bacteria and toxins to extraintestinal organs, which further mitigates immune dysregulation in the body.

The gut microbiota and its metabolites activate the immune system through multiple pathways. By producing molecules with immunoregulatory and anti-inflammatory properties, such as SCFAs, indoles, and secondary bile acids, the gut microbiota modulates immune cells, including T cells, B cells, dendritic cells, and macrophages, thereby facilitating antigen presentation and immune modulation. Specifically, SCFAs enhance Th1 cell production of IL-10 via G protein-coupled receptor 43 (GPR43) (105) and stimulate IL-22 production by cluster of differentiation (CD)4^+^ T cells and innate lymphoid cells through GPR41 and histone deacetylase inhibition (106). Secondary bile acids interact with Takeda G protein-coupled receptor 5 to reduce nucleotide-binding oligomerization domain-like receptor family pyrin domain-containing-3 inflammasome activation and downregulate proinflammatory cytokine production in macrophages by inhibiting NF-κB signaling. They also suppress NF-κB-dependent inflammatory mediator expression in macrophages through interaction with the nuclear receptor farnesoid X receptor (107). Through polysaccharide A, *Bacteroides fragilis* induces T helper (Th1) cell development and promotes immune tolerance by interacting with Toll-like receptor 2 and T cells, inhibiting Th-17 differentiation, and enhancing regulatory T-cell activity (108). Immune cells recognize microbe-associated molecular patterns, such as LPS, peptidoglycan, and flagellin, and microbiota-derived metabolites that can translocate from the gut into the systemic circulation, thereby triggering immune responses (109, 110).

For other bacterial traits such as *Dialister*, *Victivallales*, *Lentisphaerae*, *Terrisporobacter*, and *Victivallis*, the mechanisms underlying their role in the onset and progression of sepsis remain unclear due to the lack of relevant research or the existence of greater controversy. Further exploration is needed to shed light on these aspects.

The gut microbiota can be regulated by several potential prevention and treatment strategies (111). First, potential pathogens can be eradicated through selective decontamination of the digestive tract. Second, beneficial bacteria or microbe-derived metabolites can be substituted using probiotics, prebiotics, or synbiotics. Finally, the gut microbiota can be partially replaced by FMT.

Our MR analysis revealed the protective effects of *Lentisphaerae*, *Victivallales*, *Lachnospiraceae*, *Victivallis*, *Ruminiclostridium*, *Dialister*, *Coprococcus*, and *Anaerostipes* and the harmful effects of *Tenericutes*, *Bacteroidia*, *Gammaproteobacteria*, *Mollicutes*, *Bacteroidales*, *Clostridiaceae*, *Ruminococcaceae UCG 011*, *Terrisporobacter*, *Sellimonas*, and *Ruminococcus torques group* on sepsis. However, the effect of these bacterial traits in the gut microbiota on the onset and progression of sepsis has remained unclear until recently, which is limited by the current research.

Our MR study has several advantages. First, our study analyzed the causal effect of the gut microbiota on sepsis from the genus to the phylum level. This contributes to understanding the mechanisms and interactions between the gut microbiota and host immunity and facilitates the comprehensive assessment of the influence of various bacterial traits. Second, we performed MR analysis to explore the causal association between the gut microbiota and sepsis, effectively eliminating confounding factors and reverse causation, which may interfere with causal inference. Third, the genetic variants of the gut microbiota were sourced from the most extensive GWASs to date, which enhances the credibility of our findings.

Nonetheless, this MR study has limitations. First, although this study pinpointed causal associations from exposure to outcomes, it may not have accurately gauged the association’s magnitude. Further research is needed to validate these findings. Second, the use of multiple statistical corrections could be overly stringent and conservative, which might lead to overlooking bacterial traits that could have a causal association with sepsis. Therefore, with biological plausibility in mind, we did not consider multiple testing results. Third, although the majority of the participants whose gut microbiota data were collected in our study were of European descent, a small amount of the microbiological data were from other races, which may have confounded our estimates to some extent. Fourth, we opted for a less strict threshold (*p* < 1×10**^−^** ^5^) to perform horizontal pleiotropy examination and sensitivity analysis. Although this approach allowed us to identify a wider range of associations, it also increased the potential for detecting false positives. Increasing the sample size could increase the precision of the estimation of associations between the gut microbiota and sepsis. Finally, owing to the lack of individual data, we were unable to conduct further population stratification studies (e.g., gender) or explore possible differences in different populations.

## 5 Conclusion

In summary, the results of our study support the theory that the gut microbiota traits identified in this MR have a causal impact on the risk of sepsis, the risk of sepsis requiring critical care, and the 28-day mortality rate for sepsis and sepsis requiring critical care. This MR analysis could offer pioneering insights for the development of innovative prevention and treatment strategies against sepsis.

## Data availability statement

The original contributions presented in the study are included in the article/**Supplementary Material**. Further inquiries can be directed to the corresponding author.

## Ethics statement

Ethical approval was not required for the study involving humans in accordance with the local legislation and institutional requirements. Written informed consent to participate in this study was not required from the participants or the participants’ legal guardians/next of kin in accordance with the national legislation and the institutional requirements.

## Author contributions

YG: Conceptualization, Data curation, Methodology, Visualization, Writing – original draft, Writing – review & editing. LL: Writing – review & editing, Data curation, Visualization. YC: Writing – review & editing, Data curation, Visualization. JZ: Visualization, Writing – review & editing, Data curation. XW: Conceptualization, Funding acquisition, Project administration, Resources, Supervision, Writing – review & editing.
